# A Diminutive New Tyrannosaur from the Top of the World

**DOI:** 10.1371/journal.pone.0091287

**Published:** 2014-03-12

**Authors:** Anthony R. Fiorillo, Ronald S. Tykoski

**Affiliations:** Department of Paleontology, Perot Museum of Nature and Science, Dallas, Texas, United States of America; University of Pennsylvania, United States

## Abstract

Tyrannosaurid theropods were dominant terrestrial predators in Asia and western North America during the last of the Cretaceous. The known diversity of the group has dramatically increased in recent years with new finds, but overall understanding of tyrannosaurid ecology and evolution is based almost entirely on fossils from latitudes at or below southern Canada and central Asia. Remains of a new, relatively small tyrannosaurine were recovered from the earliest Late Maastrichtian (70-69Ma) of the Prince Creek Formation on Alaska's North Slope. Cladistic analyses show the material represents a new tyrannosaurine species closely related to the highly derived *Tarbosaurus*+*Tyrannosaurus* clade. The new taxon inhabited a seasonally extreme high-latitude continental environment on the northernmost edge of Cretaceous North America. The discovery of the new form provides new insights into tyrannosaurid adaptability, and evolution in an ancient greenhouse Arctic.

## Introduction

The study of tyrannosaurs, the lineage of carnivorous theropod dinosaurs that include *Tyrannosaurus rex* and its close kin has captivated the attention of the public and the scientific community since the first descriptions of these forms over a century ago [1]. The past decade has witnessed a flurry of new finds, revelations, and research on the group, swelling the ranks of recognized tyrannosaur species and fundamentally challenging long-held assumptions about the adaptability and evolution of these iconic predators [2]. For all the attention given these extinct animals, almost everything we know about them has been gathered from fossils collected at sites in the low to mid-latitudes of North America and Asia. Isolated tyrannosaurid teeth have been reported from the high latitudes of northern Alaska, but the lack of non-dental remains from the region resulted in the teeth being assigned to taxa known from much lower latitudes [3].

We report here on a new tyrannosaurid dinosaur from the North Slope of Alaska, USA. It is represented by cranial bones initially referred to the albertosaurine [3] *Gorgosaurus libratus* (Late Campanian) known from southern Alberta, Canada [4]–[7]. However, subsequent work showed the age of the material, between 70 and 69 Ma [8], [9], was more congruent with the range of the albertosaurine *Albertosaurus sarcophagus* (Early to early Late Maastrichtian), also from southern Alberta [1], [10]–[12]. Additional material, combined with closer examination and comparison with other tyrannosaurids show more similarity to tyrannosaurine forms. When added to recent cladistic analyses of tyrannosauroid relationships [2], [13], the tyrannosaurine affinities of the Alaskan material was supported. The age of the taxon places it outside the temporal ranges of the North American tyrannosaurines *Daspletosaurus torosus* and *Tyrannosaurus rex*, and it also exhibits morphological features that preclude assignment to these and the potentially contemporaneous central-Asian species *Tarbosaurus bataar*. The new tyrannosaurid fills both evolutionary and paleogeographic gaps in our knowledge of the highly derived tyrannosaurine theropods. The new form inhabited the northern-most margin of Laramidia in the early Late Cretaceous, and serves to expand our understanding of tyrannosaurid adaptability and diversification.

## Methods

### Permits

All necessary permits were obtained for the described study, which complied with all relevant regulations. The specimen was collected under BLM permit number AA-86367.

### Nomenclatural Acts

The electronic edition of this article conforms to the requirements of the amended International Code of Zoological Nomenclature, and hence the new names contained herein are available under that Code from the electronic edition of this article. This published work and the nomenclatural acts it contains have been registered in ZooBank, the online registration system for the ICZN. The ZooBank LSIDs (Life Science Identifiers) can be resolved and the associated information viewed through any standard web browser by appending the LSID to the prefix “http://zoobank.org/”. The LSID for this publication is: urn:lsid:zoobank.org:pub: 3E537CBC-B7DA-42E3-9ED6-9297A3EE4569. The electronic edition of this work was published in a journal with an ISSN, and has been archived and is available from the following digital repositories: PubMed Central, LOCKSS.

### Taxonomic Definitions

We use the informal term “tyrannosaur” in reference to members of the stem-lineage Tyrannosauroidea, encompassing all theropods more closely related to *Tyrannosaurus rex* than to *Ornithomimus edmonticus*, *Troodon formosus*, and *Velociraptor mongoliensis*. The informal term “tyrannosaurid” is used in reference to members of the node-clade Tyrannosauridae, defined as *Tyrannosaurus rex*, *Albertosaurus sarcophagus*, and *Gorgosaurus libratus*, and all descendants of their most recent common ancestor [2]. Tyrannosauridae is comprised of two formal stem lineages. The informal term “tyrannosaurine” refers to members of the stem-lineage Tyrannosaurinae which includes *Tyrannosaurus rex* and all tyrannosaurids closer to it than to *Albertosaurus sarcophagus* and *Gorgosaurus libratus*. The informal term “albertosaurine” refers to a member of the stem-lineage Albertosaurinae, which consists of *Albertosaurus sarcophagus* and *Gorgosaurus libratus*, and all tyrannosaurids closer to them than to *Tyrannosaurus rex*.Institutional Abbreviations

See Table S1 for a list of institutional abbreviations used throughout this work.

### Fieldwork and Preparation

**Figure 1:**
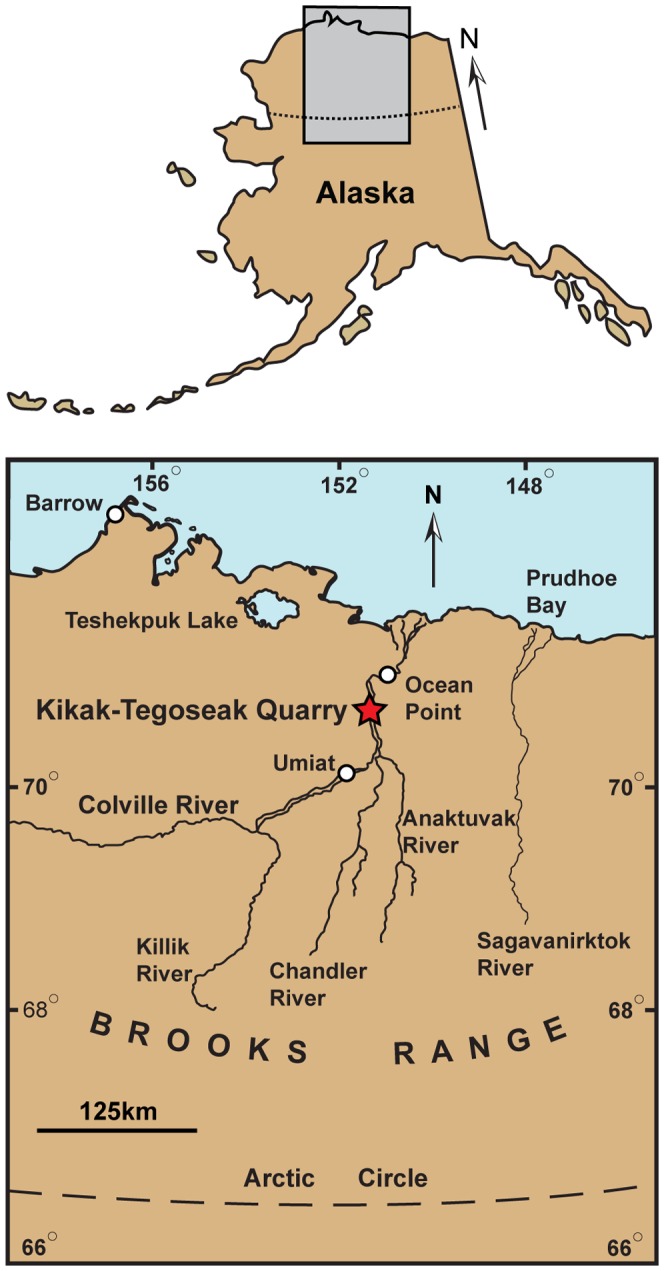
Map showing approximate location of the Kikak-Tegoseak Quarry, North Slope, Alaska, U.S.A. Gray rectangle indicates area seen in close-up view below. White circles indicate cities, towns, or settlements. Red star shows location of the Kikak-Tegoseak Quarry.

The specimens were collected in 2006 in the Kikak-Tegoseak Quarry, in an exposure of the Prince Creek Formation, North Slope Borough, Alaska, USA. (Figure 1). The specimens were originally collected in parts of three associated, but not articulated loose blocks within a square meter of one another at the edge of the quarry. The blocks were individually removed; some were wrapped in aluminum foil, then placed in sealed plastic buckets for transport out of the field area, and shipped to Dallas, Texas. Preparation was conducted in the Paleontology Lab of the Perot Museum of Nature and Science (DMNH; Dallas, Texas, USA). The specimens were extracted from the blocks using manual pneumatic tools (‘airscribes’) to remove the hard, fine-grained rock from the bones, with finer cleaning and detail work performed with sharpened tungsten-carbide points mounted in pin-vices. Broken parts were glued together using Butvar B-76 dissolved in acetone to a thick consistency. More fragile areas were treated with very dilute Butvar B-76 dissolved in acetone to allow deeper penetration of the solution into small cracks and porous areas. Molds of the specimens were made using room-temperature vulcanizing platinum-cure silicone rubber. The specimens are now housed in the collection of the Perot Museum of Nature and Science.

The partial left dentary of the specimen was also scanned using computed tomography (CT) at the University of Texas High-Resolution X-ray CT Facility at The University of Texas at Austin (Austin, Texas, USA). Scan settings and parameters were: 1024×1024 16-bit TIFF images, 450 kV, 3 mA, large spot, 1 brass filter, air wedge, 190% offset, 64 ms integration time. Slice thickness = 0.35 mm, S.O.D. 598 mm, 1200 views, 1 ray averaged per view, 2 samples per view, inter-slice spacing = 0.3 mm, field of reconstruction 240 mm (maximum field of view 240.1632 mm; field of view reported on scan form corresponds to a corrected value after applying a correction of 1.01319%; field of view equals 236.8756; reported maximum field of view reflects uncorrected value), reconstruction offset 4000, reconstruction scale 4300. Streak- and ring-removal processing based on correction of raw sinogram data using IDL routines “RK_SinoDeStreak” with default parameters, and “RK_SinoRingProcSimul” with parameters “sectors = 100, nuke_inds = [455,456,457,481,482,483,484,485,491,492,493,494,495,518,519,520,521].” Post-reconstruction ring correction applied using parameters oversampling = 2, binwidth = 11, sectors = 60. Total slices = 121.

### Geological Setting and Locality Information

**Figure 2:**
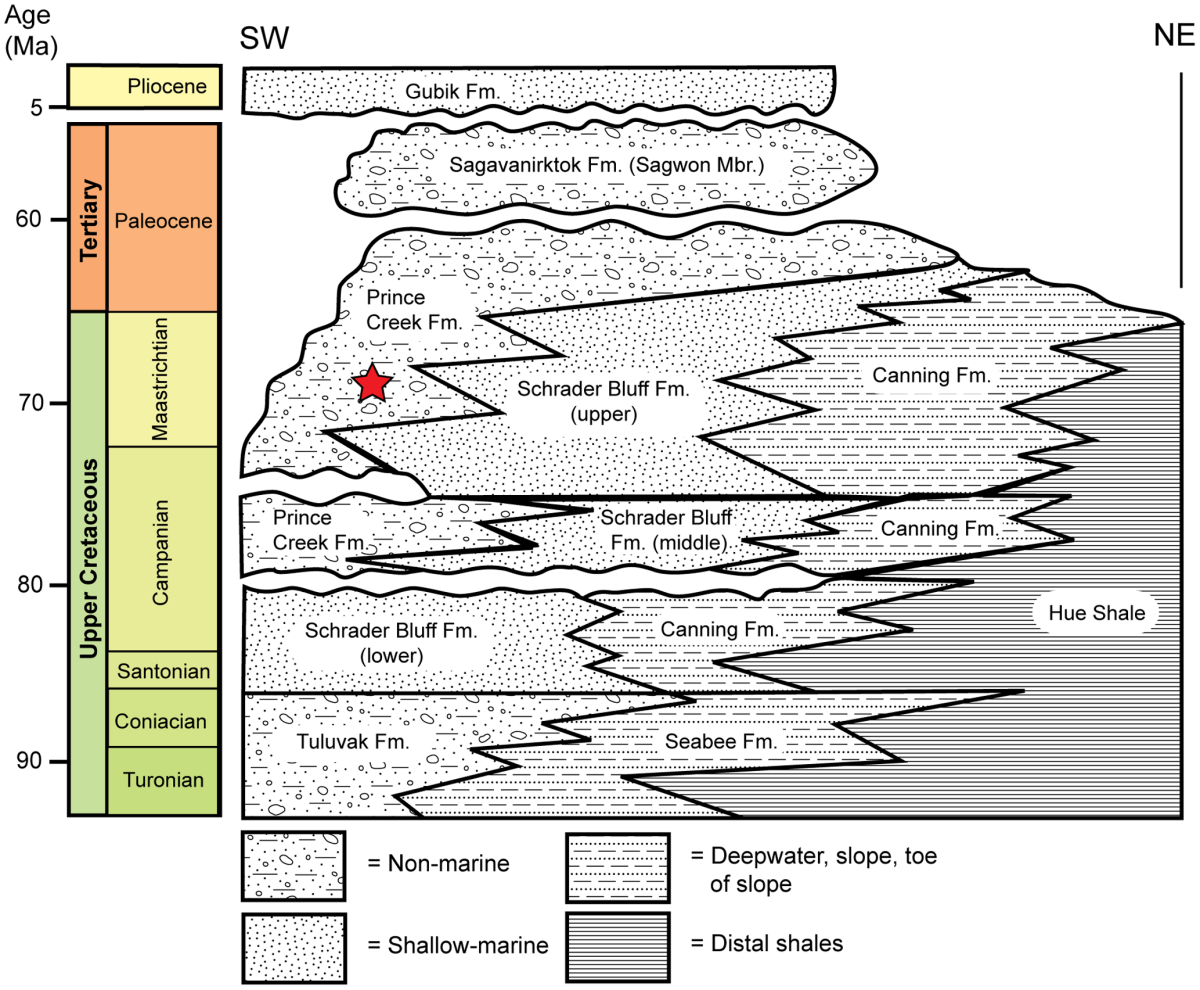
Stratigraphy as exposed along Colville River, North Slope, Alaska, USA. Approximate level of the Kikak-Tegoseak Quarry within the Prince Creek Formation is indicated by a red star.

The Kikak-Tegoseak Quarry is located in the extensive exposures of the Prince Creek Formation along bluffs bordering the Colville River, North Slope Borough, Alaska, USA. (Figure 1). The Prince Creek Formation is an alluvial unit comprised of sediments shed northward from the rising Brooks Range during late Campanian to Paleocene time (Figure 2) [8], [9]. Radioisotopic dates derived from multiple tuff beds throughout this section of the Prince Creek Formation range from 68Ma to 71Ma, with an average estimate of 69.1+/−0.3Ma, placing it in the early Late Maastrichtian [8], [9], [14]. Palynological samples from the Kikak-Tegoseak Quarry itself correlate reasonably well with the radioisotopic data, showing an Early Maastrichtian assemblage [8], [9]. More specific geographic locality information is on file at the Perot Museum of Nature and Science, in Dallas, Texas, USA.

### Systematic Paleontology

Dinosauria Owen 1852 [15] *sensu* Padian and May 1993 [16].

Theropoda Marsh 1881 [17] *sensu* Gauthier, 1986 [18].

Tyrannosauridae Osborn 1905 [1] *sensu* Holtz 2004 [19].

*Nanuqsaurus* Fiorillo and Tykoski 2014 gen. nov. urn:lsid:zoobank.org:act: 15DCBA8D-B440-49C3-9828-F89038826D76

*Nanuqsaurus hoglundi* Fiorillo and Tykoski 2014 sp. nov. urn:lsid:zoobank.org:act: 7B5B4074-9A58-4AFC-BFD0-CDCA8D799432

#### Holotype

DMNH 21461, disarticulated parts of a skull including a piece of the ascending ramus ( = nasal process, dorsal process) of the right maxilla, partial skull roof including parts of both frontals, parietals, and right laterosphenoid, and a section of the left dentary from the rostral tip to a plane through the unerupted tenth dentary tooth.

#### Etymology

*Nanuqsaurus*, combination of ‘*nanuq*’ the Iñupiaq word for polar bear (source: http://www.alaskool.org/language/dictionaries/inupiaq/dictionary.htm) and the Greek ‘sauros’ for lizard; *hoglundi*, named in recognition of Forrest Hoglund for his career in earth sciences and his philanthropic efforts in furthering cultural institutions.

#### Locality and Horizon

Kikak-Tegoseak Quarry, North Slope Borough, Alaska, USA. The site is in the Prince Creek Formation (Campanian-Paleocene) on the North Slope of Alaska [7]–[9] (Figures 1, 2). The Prince Creek Formation in this area has been dated to between around 70 and 68 million years ago, with a mean of 69.1+/−0.3 million years ago [8], [14], in early Late Maastrichtian time [10].

#### Diagnosis

A small (estimated skull length 600 mm–700 mm in mature individuals) tyrannosaurine theropod diagnosed by the following characters: thin, rostrally-forked, median spur of the fused parietals on the dorsal skull roof that overlaps and separates the frontals within the sagittal crest; frontal with a long, rostrally-pointed process separating the prefrontal and lacrimal facets; first two dentary teeth/alveoli much smaller (measured as maximum mesiodistal length) than dentary teeth/alveoli 3 through 9 in mature individuals, such that the mesiodistal length of alveolus 1 is less than 35 percent that of alveolus 3 and 25 percent or less that of alveolus 4, and such that the mesiodistal length of alveolus 2 is less than 50 percent that of alveolus 3 and 33 percent or less that of alveolus 4.

### Description

**Figure 3:**
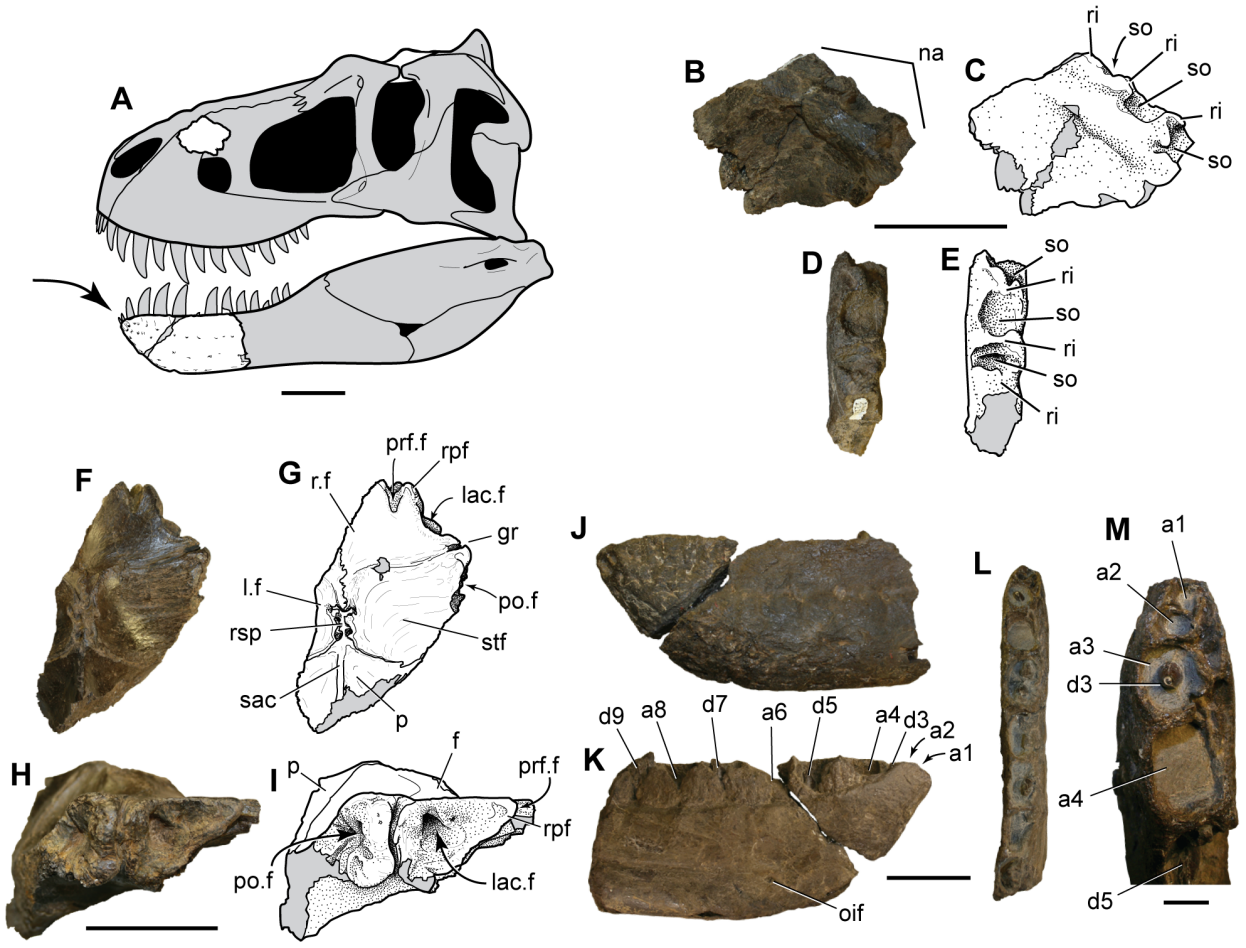
*Nanuqsaurus hoglundi*, holotype, DMNH 21461. A . Reconstruction of a generalized tyrannosaurine skull, with preserved elements of holotype shown in white. Arrow points to autapomorphic, reduced, first two dentary teeth. **B–E**. Photographs and interpretive line drawings of right maxilla piece in medial (**B**, **C**); and dorsal (**D**, **E**) views. **F–I**. Photographs and interpretive line drawings of partial skull roof in dorsal (**F**, **G**); and rostrolateral (**H**, **I**) views. **J–M**, partial left dentary in lateral (**J**); medial (**K**); dorsal (**L**) views; and close-up of mesial alveoli in dorsal (**M**) views. Abbreviations: a, alveolus, with number indicating position in tooth row; d, dentary tooth, with number indicating position in tooth row; f, frontal; gr, orbital groove; lac.f, lacrimal facet of frontal; l.f, left frontal; na, nasal contact surface; oif, oral intramandibular foramen; p, parietal; po.f, postorbital facet of frontal; prf.f, prefrontal facet of frontal;. r.f, right frontal; ri, ridge separating sockets in nasal articulation of maxilla; rpf, rostral process of frontal between prefrontal and lacrimal facets; rsp, rostral spur of parietal; sac, sagittal crest; so, socket for nasal articulation of maxilla; stf; supratemporal fossa. Gray fill indicates missing bone or broken bone surfaces and cracks. Scale bar in A equals 10 cm. Scale bars in B–L equal 5 cm. Scale bar in M equals 1 cm.

The specimen, DMNH 21461, consists of three separate pieces of a skull found in very close proximity to one another, including part of the right maxilla, a section of skull roof and braincase, and the rostral part of the left dentary. Measurements and the landmarks used in their calculations are shown in Figure S1. The maxilla fragment comes from the dorsal margin of the ascending ramus ( = dorsal process, or nasal process) of the maxilla (Figure 3B–D) (Table 1). The dorsomedial edge of the maxilla is marked by deep pockets separated by pronounced transverse ridges. These together formed a strong peg-in-socket articulation between the dorsal margin of the maxilla and the ventrolateral edge of the nasal. The same kind of deeply interlocking naso-maxillary contact is a well-documented character that is also present only in developmentally mature individuals of the derived tyrannosaurines *Daspletosaurus torosus*, *Tarbosaurus bataar*, and *Tyrannosaurus rex* [2], [6], [20], [21], [22]. The nasal-maxilla contact is either smoothly grooved or bears only weak scalloping in immature individuals and more basal tyrannosauroids [2], [6]. The presence of this feature in DMNH 21461 is evidence that the material also represents a developmentally mature individual.

**Table 1:** Selected dimensions of DMNH 21461, partial maxilla.

Maximum rostrocaudal length of specimen	86.4 mm
Maximum height of specimen	67.1 mm
Maximum width of specimen	23.2 mm
Socket for nasal articulation, most rostral socket (socket 1), maximum preserved rostrocaudal length	13.9 mm
Socket for nasal articulation, middle socket (socket 2), maximum rostrocaudal length	16.1 mm
Socket for nasal articulation, caudal socket (socket 3), maximum rostrocaudal length	8.6 mm
Nasal articulation socket 2, maximum mediolateral width	15.3 mm
Nasal articulation socket 3, maximum mediolateral width	12.8 mm
Ridge between nasal articulation sockets 1 and 2, minimum rostrocaudal width	4.3 mm
Ridge between nasal articulation sockets 2 and 3, minimum rostrocaudal width	3.7 mm

**Figure 4:**
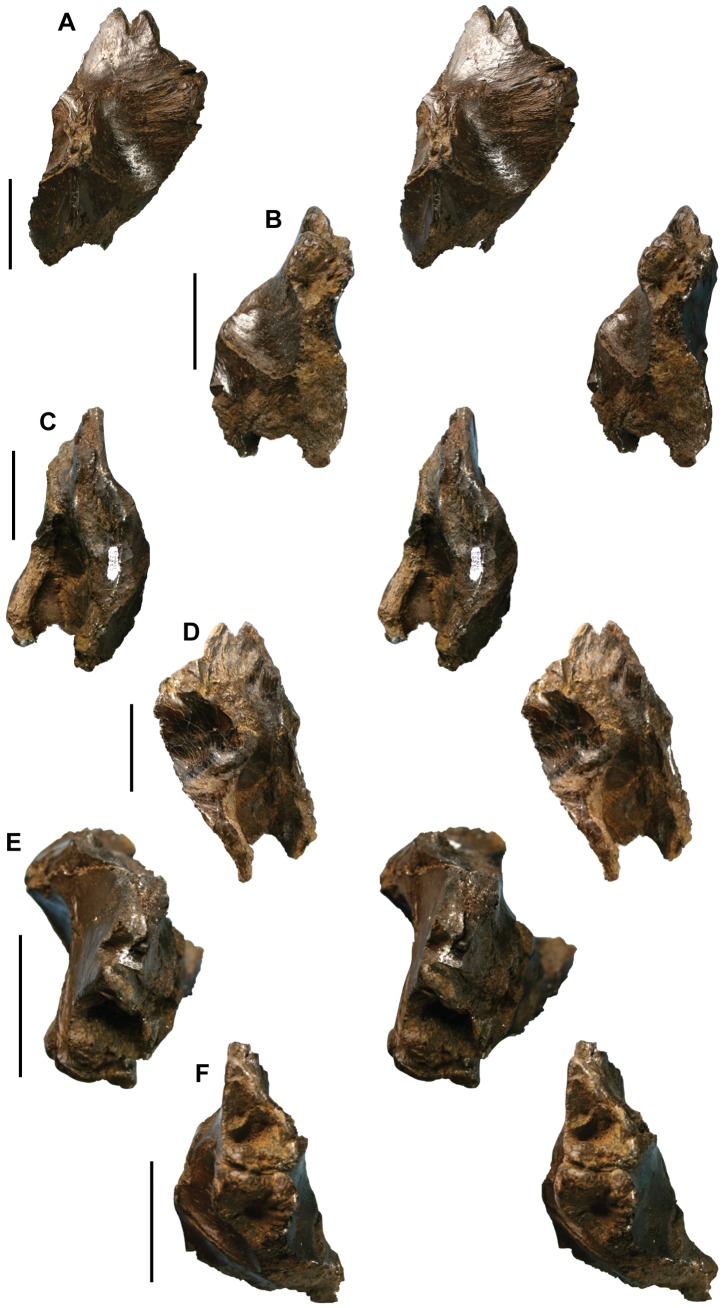
*Nanuqsaurus hoglundi*, holotype, DMNH 21461, partial skull roof piece. Stereophotographs of partial skull roof in dorsal (**A**); right lateral (**B**); left lateral (**C**); ventral (**D**); rostral (**E**); and rostrolateral (**F**) views. Rostral to top in images A through E. Scale bars equal 5 cm.

The skull roof fragment includes parts of incomplete frontals, the fused parietals, and laterosphenoid (Figures 3F–I; 4) (Table 2). The frontal has deep facets for articulation with the lacrimal rostrally and the postorbital laterally. A small notch rostromedial to the lacrimal contact is for receipt of the prefrontal. A long, rostrally-pointed process separates the notch-like facet for the prefrontal from the lacrimal facet (Fig. 3G, I). A similar process between the prefrontal and lacrimal is present in the frontal of *Teratophoneus curriei* from the upper Campanian Kaiparowits Formation of southern Utah [23], [24]. The socket-like, rostral-facing facet for the lacrimal (Figures 3H, I; 4E, F), and the process separating the prefrontal and lacrimal differ from the condition reported in the contemporaneous albertosaurine *Albertosaurus sarcophagus* from the Horseshoe Canyon Formation of southern Alberta [25].

**Table 2:** Selected dimensions of DMNH 21461, partial skull roof and braincase.

Maximum preserved length of specimen	146.2 mm
Maximum preserved height of specimen	88 mm
Right frontal, maximum mediolateral width from midline suture to most lateral point of postorbital facet	65 mm
Right frontal, rostrocaudal length from most rostral broken surface to most caudal point on frontal-parietal suture	100.9 mm
Orbital groove, maximum width in dorsal view	3.6 mm
Lacrimal facet of frontal, maximum rostromedial-caudolateral width	44.8 mm
Lacrimal facet of frontal, maximum dorsoventral height	27.1 mm
Postorbital facet of frontal, maximum dorsoventral height	31.8 mm

The dorsal skull roof of tyrannosaurids has a pronounced midline sagittal crest on the parietal and the caudal part of the frontals. The sagittal crest in *Nanuqsaurus hoglundi* is narrowly pinched on the parietal, but broadens on the frontals to form a wide, double-ridged crest (Figures 3F, G; 4A, E). A narrow spur from the parietal intrudes far rostrally between the frontals to divide the sagittal crest on the midline (Figures 3F, G; 4A). Unlike in other tyrannosaurids, this spur is forked rostrally, forming a “Y”-shaped process in dorsal view. The sagittal crest on the frontals is also divided in *Tarbosaurus bataar* and *Tyrannosaurus rex*, but is a relatively low, narrow, single crest in albertosaurines and *Daspletosaurus* [21]. The supratemporal fossae ( = dorsotemporal fossae) are deeply excavated onto the caudal parts of the frontals, and the rostral margins of the fossae are marked by sharp ridges (Figure 3F, G; 4A). Both of these features are additional ontogenetically informative characters that provide further evidence for the relatively mature status of DMNH 21461 [6], [21].

The form of the rostral part of the dentary is typical of tyrannosaurids, with a slightly convex dorsal border rostrally and weakly concave margin in the mid-section (Figure 3J–M) (Table 3). The lateral surface is penetrated by distinct rows of neurovascular foramina, one in the dorsal half of the surface, another along the ventral margin of the bone, and an incomplete third row between them. All three rows converge toward the tip of the dentary, where three large and deep foramina are aligned just below the alveolar border. The medial surface is marked by a narrow Meckelian groove that curves rostrodorsally above the oral intramandibular foramen (Figure 3K).

**Table 3:** Selected dimensions of DMNH 21461, partial left dentary.

Dentary, maximum preserved length	211 mm
Dentary dorsoventral height through septum between alveoli 4 and 5	80.5 mm
Dentary dorsoventral height through septum between alveoli 8 and 9	90.5 mm
Alveolus 1, mesiodistal length	5.6 mm
Alveolus 1, labiolingual width	6.9 mm
Alveolus 2, mesiodistal length	7.5 mm
Alveolus 2, labiolingual width	7.9 mm
Alveolus 3, mesiodistal length	16.5 mm
Alveolus 3, labiolingual width	14.7 mm
Alveolus 4, mesiodistal length	23.2 mm
Alveolus 4, labiolingual width	16.9 mm
Alveolus 5, mesiodistal length	26.7 mm
Alveolus 5, labiolingual width	13.2 mm
Alveolus 6, mesiodistal length	27.5 mm
Alveolus 6, labiolingual width	11.3 mm est.
Alveolus 7, mesiodistal length	25.4 mm
Alveolus 7, labiolingual width	12 mm
Alveolus 8, mesiodistal length	23.4 mm
Alveolus 8, labiolingual width	11.3 mm
Dentary tooth 9, maximum mesiodistal length	22.0 mm
Dentary tooth 9, maximum labiolingual width	11 mm
Rostral-most neurovascular foramen at tip of dentary, maximum mediolateral width	6.3 mm
Second most rostral neurovascular foramen at tip of dentary, maximum rostrocaudal width	6.8 mm
Oral intramandibular foramen, maximum rostrocaudal width	6.1 mm

**Figure 5:**
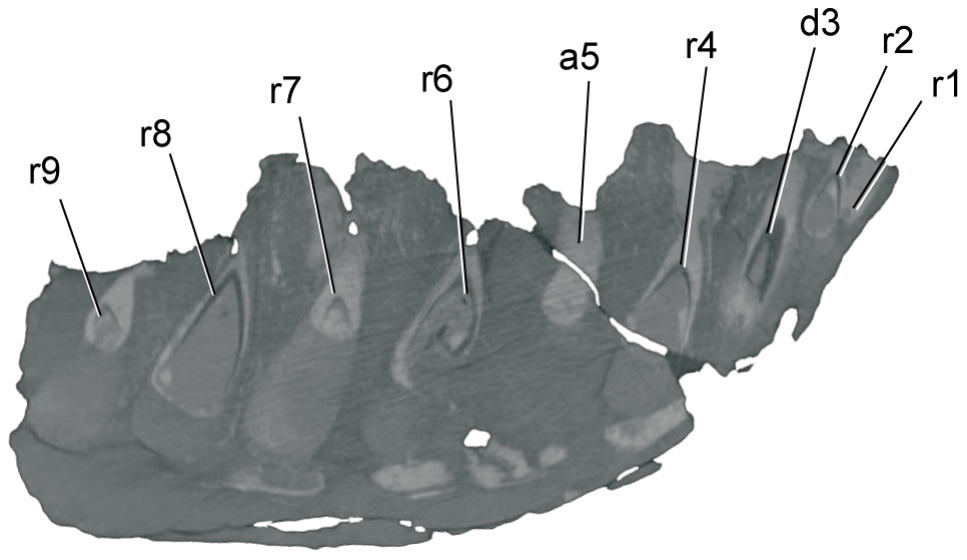
*Nanuqsaurus hoglundi,* holotype DMNH 21461, CT slice through partial left dentary. Parasagittal CT scan slice through the partial left dentary shows several replacement teeth remaining in alveoli. This slice shows parts of cross-sections through replacement teeth in the first and second alveoli, verifying their identity. Abbreviations: a, alveolus, with number indicating position in tooth row; d3, base of partially erupted third dentary tooth, r, replacement tooth (unerupted), with number indicating position in tooth row.

The preserved section of dentary has nine complete alveoli and is broken through the tenth, exposing the cross-sections of a tooth root and a small replacement tooth (Figure 3L). The exposed apices of tooth crowns are visible in the third, fifth, and seventh alveoli, and the base of a broken tooth crown is present in the ninth alveolus. The first three alveoli are nearly circular in form, the fourth is sub-rectangular, and the fifth through ninth alveoli are mesiodistally elongate and rectangular in dorsal view. The first two alveoli are very small (maximum mesiodistal lengths of 5.6 mm and 7.5 mm respectively), less than half the size of the third alveolus (16.5 mm), and less than a third that of the fourth alveolus (23.2 mm) (Figure 3M) (Table 3). The two alveoli are similar in size to the largest neurovascular foramina on the lateral surface of the dentary. CT (Computed Tomography) scans of the specimen show un-erupted teeth in both alveoli, confirming their identification (Figure 5).

The tip of the third tooth crown has a sub-circular cross-section (length to width ratio of 1.08), in contrast to the crown of the seventh dentary tooth which is labiolingually narrower (length to width ratio of 1.54). In addition, the serrated carinae on the distal teeth are on directly opposite mesial and distal margins of the tooth crown, but the mesial carina is slightly more lingually positioned on the third dentary tooth crown. In most other tyrannosaurids, only the first dentary tooth is notably reduced in size relative to the other dentary teeth, and it may also have a sub-circular cross-section with a lingually displaced lingual carina [12].

No other tyrannosaurid taxon has two reduced mesial dentary teeth, or such a size disparity between them and the more distal teeth in mature individuals as is seen in the holotype of *Nanuqsaurus hoglundi* (Figure 3A, L, M). The first two dentary teeth are reduced in the controversial tyrannosaurid specimens CMNH (Cleveland Museum of Natural History, Cleveland, Ohio, USA) 7541 and BMR (Burpee Museum of Natural History, Rockford, Illinois, USA) P2002.4.1, both of which have been identified as either a separate tyrannosaurid taxon (*Nannotyrannus lancensis*), or as ontogenetically immature *Tyrannosaurus rex* [6], [21], [25]–[28]. However, the mesiodistal length of the second dentary tooth or alveolus is still more than half the size of the third tooth/alveolus in these disputed specimens, versus approximately 45 percent in the holotype of *Nanuqsaurus hoglundi*. A very small individual attributed to *Gorgosaurus libratus* (TMP [Royall Tyrrell Museum of Palaeontology, Drumheller, Alberta, Canada] 1986.144.1) also has two small mesial dentary alveoli, but even in this immature individual the second alveolus is more than half (52 percent) the size of the third alveolus. The occurrence of two small mesial dentary teeth is not seen in all immature tyrannosaurids. A very immature specimen of *Tarbosaurus bataar* (MPC [Mongolian Paleontological Center, Ulaanbaatar, Mongolia]-D 107/7) has a well-preserved skull only 290 mm long [29]. CT scans of the specimen [29] show a relatively small first dentary alveolus and tooth, but the second alveolus/tooth is comparable in mesiodistal length (approximately 75 percent) to the third alveolus.

The condition of having two relatively small mesial dentary teeth occurs in immature specimens of some, but not all tyrannosaurid taxa as shown by MPC-D 107/7, the small specimen of *Tarbosaurus bataar* [29]. The size disparity between the mesial two versus the more distal dentary alveoli is even greater in *Nanuqsaurus hoglundi* than what is seen in immature specimens of other tyrannosaurid taxa, yet the holotype of *N. hoglundi* exhibits features of the maxilla and dorsal skull roof bones that are associated with more osteologically mature tyrannosaurid individuals [6], [21]. We therefore consider the presence of unusually small first and second dentary alveoli (and the teeth that would occupy them) in *Nanuqsaurus hoglundi* to be an autapomorphic dental character present in mature individuals of this taxon.

### Phylogenetic Analysis

There is currently only a limited amount of *Nanuqsaurus hoglundi* fossil material, but enough information is preserved to test the relationships of the taxon within the framework of two recent cladistic analyses of tyrannosauroid phylogeny [2], [24].. We first tested relationships of *N. hoglundi* in the context of a very tyrannosauroid-focused taxon-character matrix [2]. In addition to adding *N. hoglundi* to the analysis, we altered the original dataset in three ways. First, we changed the coding of two characters for *Dryptosaurus* to match revisions made in a more recent analysis of tyrannosauroid phylogeny (character 178 changed from “?” to “0”; character 201 re-scored from “?” to “0”) [30]. Second, we removed *Raptorex kreigsteini* from the analysis because of uncertainty over whether it represents a distinct tyrannosauroid taxon from the Lower Cretaceous, or is instead an immature individual of the Upper Cretaceous tyrannosaurine *Tarbosaurus bataar* [31]. The presence or absence of *Raptorex* in the analysis had no impact on the resulting tree topology. Third, we added one new character to the dataset (character 308) to take into account the shared presence of a long rostral process of the frontal inserted between the articulations for the prefrontal and lacrimal in *Nanuqsaurus* and *Teratophoneus*. These changes, plus the coding of character 308 for the other taxa in the analysis are provided in Text S1.

**Figure 6:**
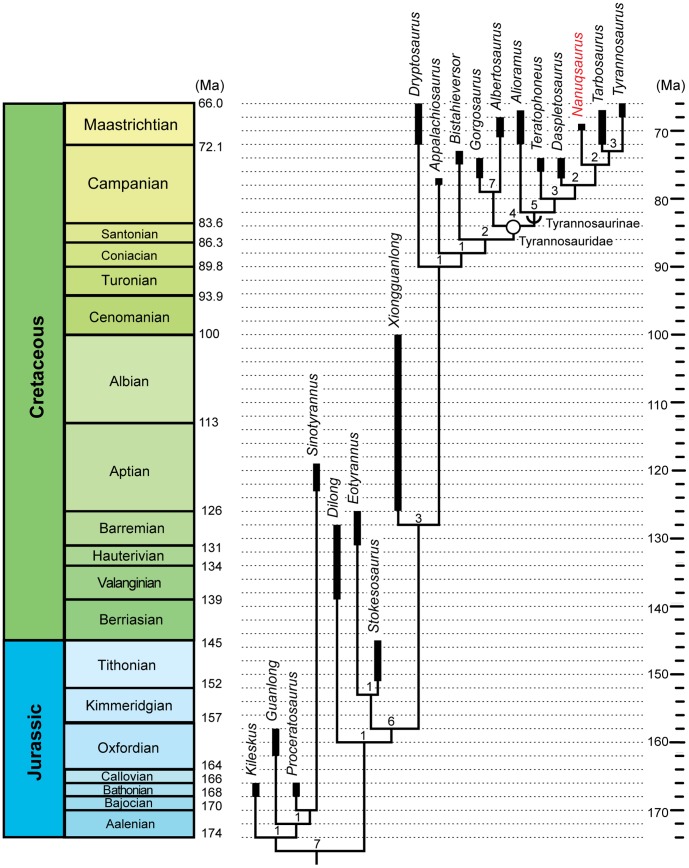
Phylogenetic position of *Nanuqsaurus hoglundi* among tyrannosauroids recovered from first phylogenetic test. One of three equally most parsimonious hypotheses of tyrannosauroid phylogeny (length = 550; consistency index = 0.6527; retention index = 0.8403) recovered in our analysis based on dataset of Brusatte et al. [2], and set to the geologic timescale. Thick bars indicate uncertainty of geological range for a given taxon. Numbers at nodes are Bremer support values for that clade. Open circle indicates node-defined clade name. Half-circle arc indicates a stem-defined lineage name. Dates for geologic stage boundaries from [10].

The original dataset [2] was acquired as a Microsoft Word document, and was reentered in MacClade 4.08a for Macintosh OSX [32]. All other characters remained numbered the same as in the original dataset, and we refer interested readers to the original study for the full character list and original dataset [2]. *Nanuqsaurus* and the aforementioned new character were added to the taxon-character matrix, resulting in 23 taxa (4 outgroup taxa, 19 ingroup taxa) and 308 characters. Character coding for *Nanuqsaurus* is given in Table S2. The dataset was subjected to a parsimony analysis in PAUP* 4.0b10 [33], using TBR branch swapping. The analysis produced three equally most parsimonious trees with a length of 550, a consistency index of 0.6527, and a retention index of 0.8403 (Figure 6). The three recovered trees differed only in the positions of *Guanlong*, *Proceratosaurus*, and *Sinotyrannus* relative to one another. Bremer support values were calculated using MacClade to create a PAUP Decay Commands file, which was then opened and applied in PAUP*.

In all three hypotheses of phylogeny, *Nanuqsaurus hoglundi* was found to be a derived tyrannosaurine, the sister taxon to the *Tarbosaurus*+*Tyrannosaurus* clade (Figure 6). This node was supported by a single unambiguous character, the presence of a dorsoventrally tall, paired sagittal crest on the frontal. The age of *Nanuqsaurus hoglundi* (70-69Ma) is consistent with its place in the recovered hypothesis of tyrannosauroid phylogeny, positioned in time between the more basal *Daspletosaurus torosus* (Middle to Late Campanian) and the more derived *Tyrannosaurus rex* (latest Maastrichtian) among other North American tyrannosaurines. *Nanuqsaurus hoglundi* may be contemporaneous with *Tarbosaurus bataar* (Late Campanian to Maastrichtian) of Asia, but the two taxa are easily distinguished from one another by the rostral process of the frontal separating the prefrontal and lacrimal in *N. hoglundi*, and by the two reduced mesial dentary teeth in *N. hoglundi* versus a single reduced first dentary tooth in juveniles and mature *T. bataar* [20], [31]. The presence of a robust peg-in-socket articulation between the maxillae and nasals of mature individuals was a character shared by *Daspletosaurus*, *Nanuqsaurus*, and the *Tarbosaurus*+*Tyrannosaurus* clade, but the uncertain status of this character in the more basal tyrannosaurines *Teratophoneus* and *Alioramus* made the position of this character ambiguous in our results. It took no less than six additional steps to move *Nanuqsaurus* to a position on the albertosaurine lineage, highlighting the low likelihood that the Alaskan material might actually be referable to the contemporaneous taxon *Albertosaurus sarcophagus*.

Although the Bremer support for the *Nanuqsaurus*+(*Tarbosaurus*+*Tyrannosaurus*) node is less (Bremer support value = 2) than that for some other nodes on the tyrannosaurine lineage (Bremer support values range from 2 to 5), given the high percentage of missing data for *Nanuqsaurus* (95.1 percent missing data) its location on the tree is an indication of the importance of the derived characters present in the material. Indeed, there is less support for the positions of the more basal tyrannosauroids *Appalachiosaurus* and *Dryptosaurus*, both of which are known from more material than *Nanuqsaurus*. Bremer support for other tyrannosaurine nodes was less in our analysis than was recovered in the study upon which it was based [2]. In the original study, the *Tarbosaurus*+*Tyrannosaurus* clade had a very high Bremer support value of 9, and the *Daspletosaurus*+(*Tarbosaurus*+*Tyrannosaurus*) clade a very high Bremer value of 7. The dramatic reduction in Bremer values in our analysis likely reflects the intermediate position of *Nanuqsaurus* in the hypotheses of phylogeny, which serves to break up or redistribute potential diagnostic characters among the derived tyrannosaurines.

The second cladistic test was based on that used in a more recent study that included a greater number of taxa from across a much wider range of theropod clades [24]. The dataset from the original study also used a large number of new and altered characters compared to other recent studies [2], [13], [24]. Not surprisingly then, the hypotheses of tyrannosauroid relationships generated by the newer work differed from previous studies. Most notably, the basal tyrannosaurine *Alioramus* was placed outside Tyrannosauridae, and *Bistahieversor*, *Teratophoneus*, and the newly described *Lythronax argestes* from the Middle Campanian of Utah were found to be members of the tyrannosaurine lineage more derived than *Daspletosaurus torosus* [24].

We added *Nanuqsaurus hoglundi* to the second dataset, but made no other changes to the original taxon-character matrix. The matrix was copied directly from an electronic version of the paper into Microsoft Word, and then imported into Mesquite v.2.75 [34]. Character coding for *Nanuqsaurus* in the second analysis is given in Table S3. The file was then opened in MacClade 4.08a for Macintosh OSX [32], and finally was subjected to a parsimony analysis in PAUP* 4.0b10 [33], using TBR branch swapping. The analysis recovered eight equally most parsimonious trees, each with a length of 2008 evolutionary steps, a consistency index of 0.3541, and a retention index of 0.7226. Bremer support values were calculated using MacClade to create a PAUP Decay Commands file that was opened and applied in PAUP*.

**Figure 7:**
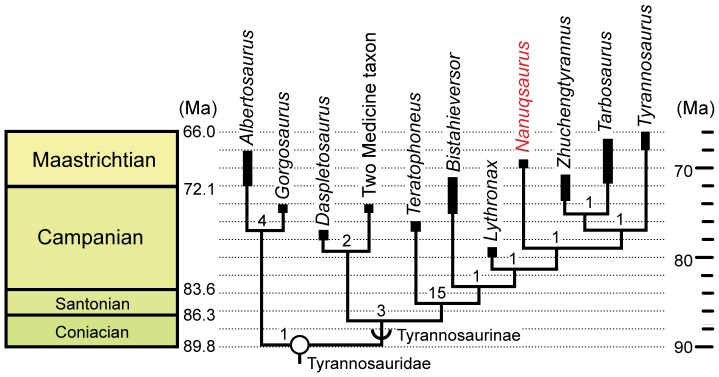
Phylogenetic position of *Nanuqsaurus hoglundi* within Tyrannosauridae recovered from second phylogenetic test. Part of one of eight equally most parsimonious hypotheses of tyrannosauroid phylogeny (length = 2008; consistency index = 0.3541; retention index = 0.7226) found in our analysis based on dataset of Loewen et al. [24], and set to the geologic timescale. Figure shows only the relationships within Tyrannosauridae. Thick bars indicate uncertainty of geological range for a given taxon. Numbers at nodes are Bremer support values for that clade. Open circle indicates node-defined clade name. Half-circle arc indicates a stem-defined lineage name. Dates for geologic stage boundaries from [10].

In addition to greater tree length, the hypotheses of relationship in our second analysis differed from the original work [24] in several other ways. These included the removal of *Dryptosaurus* from Tyrannosauroidea, additional altered relationships among basal tyrannosauroids, and non-tyrannosaurid taxa, and a sister-taxon relationship between *Daspletosaurus torosus* and the Two Medicine Formation taxon. Importantly, *Nanuqsaurus hoglundi* was again found to be a derived tyrannosaurine, and the sister-taxon to the (*Tyrannosaurus*+(*Tarbosaurus*+*Zhuchengtyrannus*)) clade in all eight recovered trees. The relationships within Tyrannosauridae generated by the second analysis are shown in Figure 7.

## Results and Discussion

### Laramidian Geographic Provincialism

The North American continent was divided by the Western Interior Seaway during the Late Cretaceous, resulting in the eastern landmass Appalachia, and the western landmass Laramidia [35]. Paleogeographic and tectonic reconstructions indicate Laramidia was a latitudinally elongate, but narrow landmass that extended from modern-day central Mexico to the Arctic Ocean on the Alaskan north coast. Recent works and discoveries of new ceratopsid dinosaur taxa have provided support for hypotheses that Laramidian dinosaur communities were divided into northern and southern provinces in the Late Campanian by topographic complexity, resulting in increased dinosaur diversity along the narrow band of fluvial plains and coastal regions bordering the Western Interior Seaway [36]–[39]. Regression of the seaway began in Maastrichtian time, eventually re-linking the North American landmasses and apparently reducing provincialism in the dinosaurian fauna [38].

The discovery of a new tyrannosaurine theropod on the extreme northern margin of Laramidia may be further evidence of the importance of topographic complexity driving dinosaur diversity during the Late Cretaceous. Is it reasonable to expect significant dinosaur diversification in the Cretaceous Alaskan Arctic, and why? Vertebrates can show striking diversification patterns resulting from a variety of geographic barriers, as opposed to ecological factors such as local net primary productivity, a biogeographic model that has successfully explained biodiversity in modern and fossil vertebrates in continental and aquatic environments [40]–[43].

There is geological evidence that the coastal plain of Laramidia was a relatively heterogeneous landscape driven by orogenic episodes during the Cretaceous [42]. The sediments of the Prince Creek Formation were deposited in the east-west trending Colville Basin, which formed by the collision of the Arctic Alaska plate with the plates comprising central Alaska [44]. The collision of these plate boundaries also resulted in formation of the Brooks Range to the south of the Colville Basin, which may have been up to 5000 meters in height during the Cretaceous [45]. Mountain building has been suggested as the trigger for diversification of Late Cretaceous herbivorous dinosaurs in lower latitudes [42]. Given the existence of a unique ceratopsid species also from the same locality as *Nanuqsaurus hoglundi*, it is possible that an effective geographic barrier may have been provided by the ancestral Brooks Range [7] creating, effectively, an island within the larger landmass of Laramidia. The presence of a new tyrannosaurine theropod from the same region is consistent with this biogeographic model of vertebrate diversification.

### Body Size of *Nanuqsaurus*

**Figure 8:**
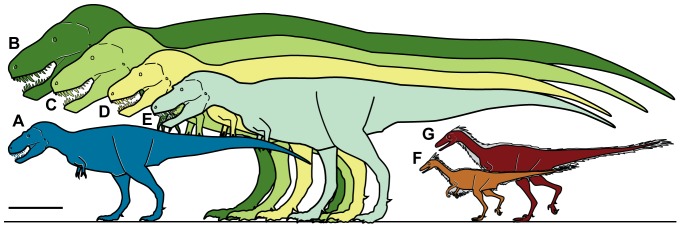
Relative size of *Nanuqsaurus hoglundi*. Silhouettes showing approximate sizes of representative theropods. **A**, *Nanuqsaurus hoglundi*, based on holotype, DMNH 21461. **B**, *Tyrannosaurus rex*, based on FMNH PR2081. **C**, *Tyrannosaurus rex*, based on AMNH 5027. **D**, *Daspletosaurus torosus*, based on FMNH PR308; **E**, *Albertosaurus sarcophagus*, based on TMP 81.10.1; **F**, *Troodon formosus*, lower latitude individual based on multiple sources and size estimates; **G**, *Troodon* sp., North Slope individual based on extrapolation from measurements of multiple dental specimens [47]. Scale bar equals 1 m.

Although northern Alaska was warmer than present during the Cretaceous [8], [9], [45], [46] the region still experienced profound seasonal changes in light regime through the course of a solar year [45], [46]. Many extant vertebrates live in regions of the world with a seasonal climate resulting in a non-growing season, and those organisms depend on the amount of resources acquired during the growing season. Modern vertebrates deal with these conditions through behavioral, physiological, or phenotypical changes in contrast to related forms in less seasonal regimes. Evidence shows that dinosaurian taxa may have responded similarly to these conditions. For example, Alaskan individuals of the otherwise small theropod *Troodon* were shown to have achieved body sizes approximately 50% larger than specimens from southern Alberta and Montana [47] (Figure 8). The increased size was attributed to the larger eyes in proportion to body size of *Troodon* with respect to sympatric theropods, which provided a competitive advantage in accessing more prey items in the low-light conditions of the ancient Arctic.

Conversely, the estimated skull length of *Nanuqsaurus hoglundi* suggests that this animal was of smaller size than coeval and related tyrannosaurids from the lower latitudes (Figure 8). Tyrannosaurs are notable by, among other things, large body size near the upper limits of theropods as a whole. Derived tyrannosaurs are also recognized as having enhanced olfaction [48]. Their large body size suggests the animals were active hunters, rather than scavengers [49]. With respect to extant carnivores, the representatives at the upper limits of each group are constrained by food intake [50]. The smaller body size of *Nanuqsaurus hoglundi* may reflect that in the profoundly seasonal ancient Arctic environment where the widely varying light regime affected biological productivity; resource availability was limited. The phenotypic response by the apex predator within this ecosystem was smaller body mass, a pattern similar to that observed with modern carnivores in an insular setting where resources are also limited compared to resource availability for mainland populations [51].

The increase in body size by one theropod taxon and the reduction in body size by a second theropod taxon suggest that there may have been external ecological pressure for an optimal body size for predatory dinosaurs in the seasonal Cretaceous Arctic ecosystem of northern Alaska. This convergence in body size was dictated by the effective net biological productivity during the growing season. For *Nanuqsaurus hoglundi* the resource limits selected for smaller body size, while for the sympatric *Troodon* the adaptive advantage of larger eyes in the highly seasonal physical environment selected for larger body size. Niche separation however was maintained by reliance on different senses to acquire food items. Further, this body size response likely reflects that the role of body size in these predatory dinosaurs was key in the structuring of the ecosystem. Therefore understanding polar theropods is crucial to understanding potential feedback loops between dinosaurs and climate in the ancient Arctic.

## Conclusions

Tyrannosaurid theropods were dominant, apex terrestrial predators in Asia and western North America during Campanian and Maastrichtian time. Knowledge of the group's diversity has increased in recent years as a result of new discoveries. Overall understanding of tyrannosaurid ecology and evolution is based almost entirely on fossils from latitudes at or below southern Canada and central Asia. The discovery of the new, relatively small tyrannosaurid *Nanuqsaurus hoglundi* from the earliest Late Maastrichtian (70-69Ma) section of the Prince Creek Formation on Alaska's North Slope provides new information regarding the diversity, distribution, and adaptability of derived tyrannosaurs.

Two phylogenetic analyses found *Nanuqsaurus hoglundi* was a tyrannosaurine and the sister-taxon to the highly derived *Tarbosaurus*+*Tyrannosaurus* clade. Although the preserved elements of *N. hoglundi* are admittedly fragmentary (a partial skull roof, maxilla and dentary), there are enough morphological data preserved to provide good support for its place among derived tyrannosaurines. *Nanuqsaurus hoglundi* is further distinguished from all contemporaneous tyrannosaurids by: 1) a narrow, median, forked spur of the parietal that overlaps the frontal dorsally between the divided sagittal crest on the frontal; 2) a long, rostrally-pointed process of the frontal separating the prefrontal and lacrimal facets; and 3) the first two dentary teeth/alveoli less than one-half the mesiodistal length of the third dentary tooth/alveolus in relatively mature individuals.

*Nanuqsaurus hoglundi* inhabited a high-latitude continental environment on the northernmost edge of Cretaceous North America. Conditions in this setting were relatively warm, but there were profound to extreme seasonal changes in light regime throughout the year that would have limited resource availability and produced substantial variance in temperatures. This northern fringe of western North America (Laramidia) also may have been partially isolated from southern regions by the topographic barrier created by the east-west trending Brooks Range, providing conditions for increased dinosaur diversification. The resource limitations of this environment seem to have selected for an optimal body size among predatory dinosaurs that was expressed by the diminutive, mature body size of *N. hoglundi* when compared to most tyrannosaurid taxa known from lower latitudes. The discovery of *Nanuqsaurus hoglundi* provides new insights into tyrannosaurid adaptability and evolution in an ancient greenhouse Arctic.

## Supporting Information

Figure S1 Reference points for measurements. Line drawings of *Nanuqsaurus hoglundi* DMNH 21461 cranial elements, illustrating the reference points used to obtain several of the measurements provided in Tables 1, 2, and 3. **A**, partial ascending ramus ( = nasal process, dorsal process) of right maxilla in medial view; **B**, partial ascending ramus ( = nasal process, dorsal process) of right maxilla in dorsal view; **C**, partial skull roof and braincase elements in dorsal view; **D**, partial skull roof and braincase elements in right rostrolateral view; **E**, partial left dentary in lateral view; **F**, close up of rostral end of dentary in dorsal view, showing reference points for measurements of first through fourth alveoli. For dimensions in F, see Table 3. All values given are in millimeters.
(TIF)

Table S1 List of Institutional Abbreviations.
(DOC)

Table S2 Character coding for *Nanuqsaurus hoglundi* in the first of two cladistic analyses. The first cladistic analysis was based on the taxon-character matrix of Brusatte et al.
(DOC)

Table S3 Character coding for *Nanuqsaurus hoglundi* in the second of two cladistic analyses. The second analysis was based on the taxon-character matrix of Loewen et al. No new characters were added to the original character list, and the original analysis was followed by treating 48 of 501 characters as ‘ordered’. Readers are referred to the original work.
(DOC)

Text S1 Changes to the taxon-character matrix in the first cladistic analysis.
(DOC)
