# Supplementary figures and images for: A Diminutive New Tyrannosaur from the Top of the World
Source: PLoS One. 2014 Mar 12;9(3):e91287. doi: 10.1371/journal.pone.0091287 (PMC3951350; doi:10.1371/journal.pone.0091287)

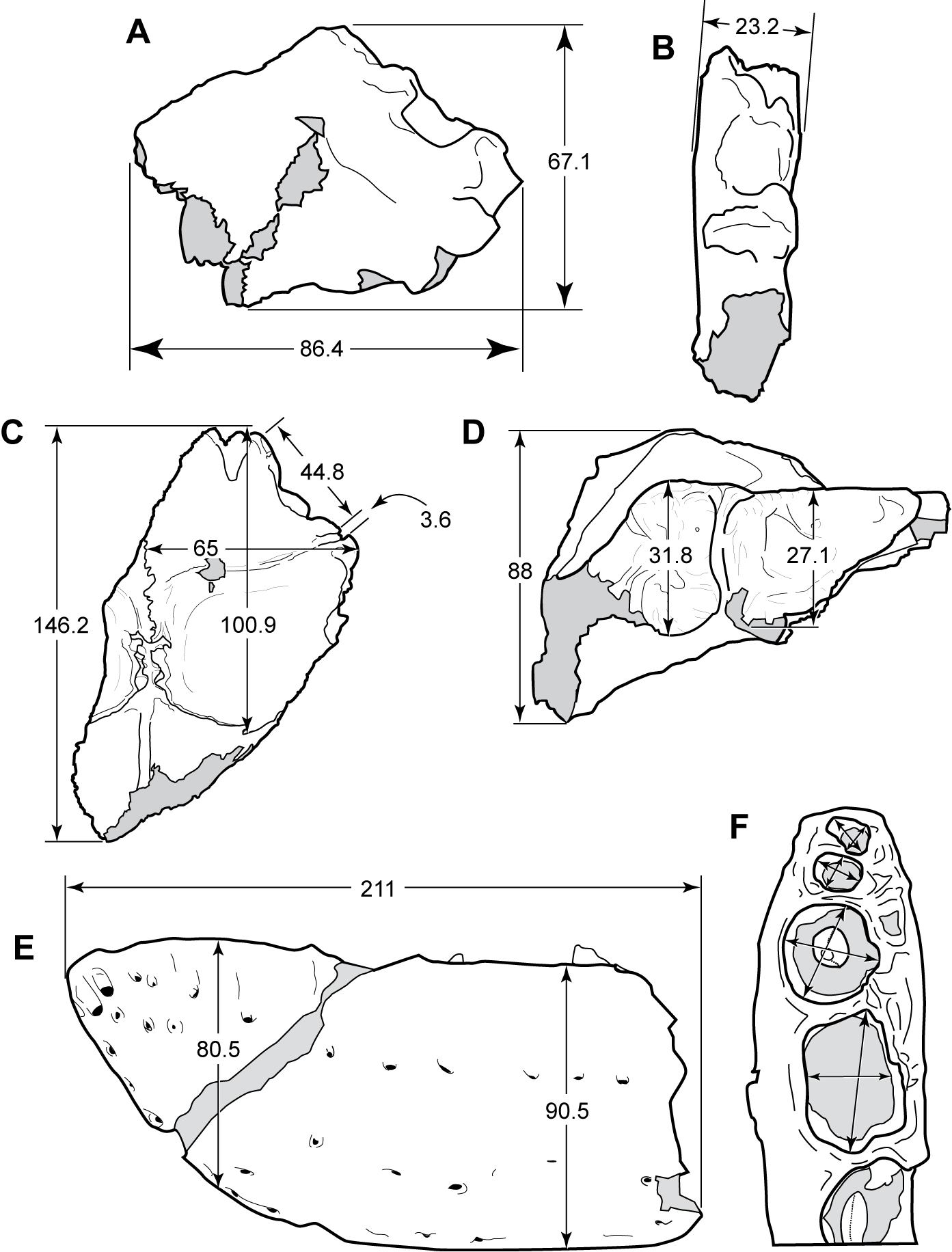

Supplement: Figure S1 — Reference points for measurements. Line drawings of Nanuqsaurus hoglundi DMNH 21461 cranial elements, illustrating the reference points used to obtain several of the measurements provided in Tables 1, 2, and 3. A, partial ascending ramus ( = nasal process, dorsal process) of right maxilla in medial view; B, partial ascending ramus ( = nasal process, dorsal process) of right maxilla in dorsal view; C, partial skull roof and braincase elements in dorsal view; D, partial skull roof and braincase elements in right rostrolateral view; E, partial left dentary in lateral view; F, close up of rostral end of dentary in dorsal view, showing reference points for measurements of first through fourth alveoli. For dimensions in F, see Table 3. All values given are in millimeters. (TIF) [file pone.0091287.s001.tif]
