# Supplementary material for: A Diminutive New Tyrannosaur from the Top of the World
Source: PLoS One. 2014 Mar 12;9(3):e91287. doi: 10.1371/journal.pone.0091287 (PMC3951350; doi:10.1371/journal.pone.0091287)
Supplement: Table S1 — List of Institutional Abbreviations. (DOC) [file pone.0091287.s002.doc]

**Table S1. List of Institutional Abbreviations.**

AMNH, American Museum of Natural History, New York, USA;

BMR, Burpee Museum of Natural History, Rockford, Illinois, USA;

CMNH, Cleveland Museum of Natural History, Cleveland, Ohio, USA;

DMNH, Perot Museum of Nature and Science, Dallas, Texas, USA;

FMNH, The Field Museum, Chicago, Illinois, USA;

MPC. Mongolian Paleontological Center, Ulaanbaatar, Mongolia;

TMP, Royal Tyrrell Museum of Palaeontology, Drumheller, Alberta, Canada.
