# Supplementary material for: A Diminutive New Tyrannosaur from the Top of the World
Source: PLoS One. 2014 Mar 12;9(3):e91287. doi: 10.1371/journal.pone.0091287 (PMC3951350; doi:10.1371/journal.pone.0091287)
Supplement: Table S2 — Character coding for Nanuqsaurus hoglundi in the first of two cladistic analyses. The first cladistic analysis was based on the taxon-character matrix of Brusatte et al. (DOC) [file pone.0091287.s003.doc]

**Table S2: Character coding for *Nanuqsaurus hoglundi* in the first of two cladistics analyses.** This analysis was based on the taxon-character matrix of Brusatte et al. [3].

*Nanuqsaurus_hoglundi* ??????????????????????????????2????????????????????????????????????????????????????????????????????????????????????1212211111?1???????????????????????????????????????????1???1?1??????????????????????????????????????????????????????????????????????????????????????????????????????????????????????????????????1
