# Supplementary material for: A Diminutive New Tyrannosaur from the Top of the World
Source: PLoS One. 2014 Mar 12;9(3):e91287. doi: 10.1371/journal.pone.0091287 (PMC3951350; doi:10.1371/journal.pone.0091287)
Supplement: Table S3 — Character coding for Nanuqsaurus hoglundi in the second of two cladistic analyses. The second analysis was based on the taxon-character matrix of Loewen et al. No new characters were added to the original character list, and the original analysis was followed by treating 48 of 501 characters as ‘ordered’. Readers are referred to the original work. (DOC) [file pone.0091287.s004.doc]

**Table S3. Character coding for *Nanuqsaurus hoglundi* in the second of two cladistic analyses.** Second analysis was based on the taxon-character matrix of Loewen et al. [25]. No new characters were added to the original character list, and the original analysis was followed by treating 48 of 501 characters as ‘ordered’. Readers are referred to the original work [25] for the list of ordered characters.

*Nanuqsaurus_hoglundi* ??????????????????????????????????????2??????????????????????????????????????????0????1??2101?12312?0????????????????????????????????????????????????????????????????????????????????1?????????????????????????????????????????????????????????0???01???1?1?110?????????????????????????????????????????????0????????????????????????????????????????????????????????????????????????????????????????????????????????????????????????????????????????????????????????????????????????????????????????????????????????
