# Supplementary material for: A Diminutive New Tyrannosaur from the Top of the World
Source: PLoS One. 2014 Mar 12;9(3):e91287. doi: 10.1371/journal.pone.0091287 (PMC3951350; doi:10.1371/journal.pone.0091287)
Supplement: Text S1 — Changes to the taxon-character matrix in the first cladistic analysis. (DOC) [file pone.0091287.s005.doc]

**Text S1. Changes to the taxon-character matrix in the first cladistic analysis.** Two separate phylogenetic analyses were performed to establish hypotheses of relationship between *Nanuqsaurus hoglundi* and other tyrannosauroids. The first analysis was based on the taxon-character matrix of Brusatte et al. [3], but differed from the original analysis in: 1) the coding of two characters for *Dryptosaurus* (character 178 changed from “?” to “0”; character 201 changed from “?” to “0”); 2) the removal of *Raptorex kriegsteini* from the analysis; and 3) the addition of one new character; Character 308. Frontal, facets for contact with prefrontal and lacrimal adjacent to one another or separated by short span of bone (0), or separated by long, rostrally-pointed process of the frontal (1). The codings for character 308 in each of the other operational taxonomic units in the analysis were:

*Allosaurus* = 0

Dromaeosauridae = 0

Ornithomimosauria = 0

Compsognathidae = 0

*Kileskus* = ?

*Guanlong* = 0

*Proceratosaurus* = ?

*Dilong* = 0

*Eotyrannus* = ?

*Stokesosaurus* = ?

*Xiongguanlong* = ?

*Dryptosaurus* = ?

*Appalachiosaurus* = ?

*Albertosaurus* = 0

*Gorgosaurus* = 0

*Alioramus* = 0

*Daspletosaurus* = 0

*Tarbosaurus* = 0

*Tyrannosaurus* = 0

*Bistahieversor* = 0

*Teratophoneus* = 1

*Sinotyrannus* = ?
